# A miniaturized multicellular platform to mimic the 3D structure of the alveolar-capillary barrier

**DOI:** 10.3389/fbioe.2024.1346660

**Published:** 2024-04-05

**Authors:** Michela Licciardello, Cecilia Traldi, Martina Cicolini, Valentina Bertana, Simone Luigi Marasso, Matteo Cocuzza, Chiara Tonda-Turo, Gianluca Ciardelli

**Affiliations:** ^1^ La.Di.Spe Bioengineerig, Politecnico di Torino, Department of Mechanical and Aerospace Engineering, Turin, Italy; ^2^ PolitoBIOMed Lab, Politecnico di Torino, Turin, Italy; ^3^ Interuniversity Center for the Promotion of the 3Rs Principles in Teaching and Research, Italy; ^4^ ChiLab- Materials and Microsystems Laboratory, Politecnico di Torino, Department of Applied Science and Technology (DISAT), Chivasso, Italy; ^5^ CNR-IMEM, National Research Council-Institute of Materials for Electronics and Magnetism, Parma, Italy; ^6^ CNR-IPCF, National Research Council-Institute for Chemical and Physical Processes, Pisa, Italy

**Keywords:** alveolar-capillary barrier, *in vitro* models, alveolus-on-a-chip, ECM-like substrate, cell tri-culture

## Abstract

Several diseases affect the alveoli, and the efficacy of medical treatments and pharmaceutical therapies is hampered by the lack of pre-clinical models able to recreate *in vitro* the diseases. Microfluidic devices, mimicking the key structural and compositional features of the alveoli, offer several advantages to medium and high-throughput analysis of new candidate therapies. Here, we developed an alveolus-on-a-chip recapitulating the microanatomy of the physiological tissue by including the epithelium, the fibrous interstitial layer and the capillary endothelium. A PDMS device was obtained assembling a top layer and a bottom layer obtained by replica molding. A polycaprolactone/gelatin (PCL-Gel) electrospun membrane was included within the two layers supporting the seeding of 3 cell phenotypes. Epithelial cells were grown on a fibroblast-laden collagen hydrogel located on the top side of the PCL-Gel mats while endothelial cells were seeded on the basolateral side of the membrane. The innovative design of the microfluidic device allows to replicate both cell-cell and cell-extracellular matrix interactions according to the *in vivo* cell arrangement along with the establishment of physiologically relevant air-liquid interface conditions. Indeed, high cell viability was confirmed for up to 10 days and the formation of a tight endothelial and epithelial barrier was assessed by immunofluorescence assays.

## 1 Introduction

Alveoli are the functional unit of the human lung in which the gas exchanges between air and blood take place at the level of the alveolar-capillary barrier that is characterized by a layered three-dimensional (3D) and multicellular structure. The alveolar unit includes two main compartments: (i) the basement membrane, a specialized extracellular matrix (ECM) membrane involved in supporting the epithelial and endothelial cell layers (Nicholas and Jacques, 2005; Nishiguchi et al., 2017) and (ii) the pulmonary interstitial space a dense fibrous network of collagen and elastin in which fibroblasts are the predominant resident cells (White, 2015; Beretta et al., 2021). The microanatomy of the alveoli is represented by the interstitial space interposed between the alveolar epithelium and the capillary endothelial layer (Varon et al., 2008).

Several diseases affect the alveoli, but the efficacy of medical treatments and pharmaceutical therapies is hampered by the lack of pre-clinical models able to recreate *in vitro* the diseases.

Over the past decades, researchers have tackled the development of advanced technologies to improve the predictive power of pre-clinical *in vitro* models which are proposed as alternative methods to animal testing. Indeed, the widely used two-dimensional (2D) models fail to fully reflect the multicellular architecture and complex microenvironment of the healthy and pathological human alveoli (Asghar et al., 2015; Yang et al., 2018) limiting their reliability to mimic the complex 3D environment of human tissues. Microfluidic systems have recently attracted strong interest thanks to the high surface-to-volume ratio and the high-throughput capability that allow for the accurate handling of biological samples (Chang et al., 2015). Lung-on-a-chip microfluidic devices have emerged as effective lung cell culture systems due to their ability to recreate the three-dimensional architecture, multicellular composition, vascular perfusion, and physiological microenvironment of native lung tissue (Bhatia and Ingber, 2014; Zhu, 2020). In literature, lung-on-a-chips with different designs have been developed, obtaining systems that resemble the native architecture and the physiological stimuli within the alveoli (Benam et al., 2016; Jain et al., 2018; Felder et al., 2019). Typically, lung-on-a-chip systems consist of multiple layers of elastomeric polydimethylsiloxane (PDMS) containing microchannels to allow the flow of cell culture medium. These layers are divided by porous PDMS or polyethylene terephthalate (PET) membranes that serve as substrate for cell culture (Hassell et al., 2017; Huh et al., 2018; Stucki et al., 2018). However, PDMS membranes are unable to recapitulate the composition, architecture, topography and stiffness of the alveolar extracellular matrix (ECM), affecting cell function and response to stimuli (Huang et al., 2021). To overcome this limitation, advanced lung-on-a-chips have been developed to accommodate ECM-like substrates (Higuita-Castro et al., 2017; Zamprogno et al., 2021; Dasgupta et al., 2023), implementing biomimetic co-culture systems of alveolar epithelial and endothelial cells. For example, Higuita-Castro et al. (Higuita-Castro et al., 2017) reported the development of a lung-on-a-chip that integrated an electrospun polycaprolactone-gelatin (PCL-Gel) membrane for mimicking the topography and composition of the basement membrane of the alveolar-capillary barrier. However, this model relies on alveolar epithelial cells cultured in non-physiologic submerged condition, which is known to hinder the cell polarization and secretion of mucus or surfactant (Doryab et al., 2019). In the work of Dasgupta et al. (Dasgupta et al., 2023), a microfluidic organ-on-a-chip was developed, establishing a dynamically perfused coculture of human lung alveolar epithelial cells cultured under an air-liquid interface (ALI) and primary human lung microvascular endothelial cells, interfacing with an ECM-coated porous PDMS membrane. The model was shown to replicate the functionality of the alveolar-capillary barrier as well as many distinctive features of radiation-induced lung injury.

However, a current limitation of reported systems is the absence of the interstitial space that prevents a reliable simulation of the thick portion of the alveolar-capillary barrier. In this regard, several studies reported the applicability of hydrogel culture models with encapsulated lung fibroblasts to reproduce lung 3D microenvironment and recapitulate lung fibroblast-ECM interactions, as reviewed by Phogat et al. (Phogat et al., 2023). Specifically, different studies suggest the use of fibroblasts embedded in 3D collagen matrices as *in vitro* mesenchymal models (Ishikawa et al., 2017), highlighting the role of ECM-like hydrogels in affecting lung phenotype and functions (Hackett et al., 2022; Phogat et al., 2023). In addition, fibroblast-laden hydrogels and epithelial cell cocultures have been used to model lung epithelial–interstitial interface (He et al., 2017; Ishikawa et al., 2017; Kang et al., 2021), as they reproduce the microenvironment of cell growth with higher physiological relevance compared to transwell-based 2D culture systems.

Moreover, many studies highlight the role of stroma in the progression of alveolar inflammatory-related disorders such as lung fibrosis (Doryab et al., 2022) and cancer (Koukourakis et al., 2017). For example, Doryab et al. (Doryab et al., 2022) established a trilayered culture using pathological cells in a transwell-like system to model lung fibrosis, recapitulating different biological hallmarks of pulmonary fibrosis.

In this context, we recently described the development of a biomimetic transwell system integrating a PCL-Gel membrane and a lung fibroblast (MRC-5) - type I collagen hydrogel lined by human microvascular endothelial cells (HULEC-5a) and alveolar epithelial cells (A549), respectively, which mimics the physiological air-liquid interface (ALI) cell culture condition as well as the tri-layered architecture and the physio-pathological events (LPS-mediates infection) occurred *in vivo* (Licciardello et al., 2023)*.* Exploiting this approach, here we report the implementation of a biomimetic microfluidic device (called alveolus-on-a-chip) to obtain a miniaturized model of the thick portion of the alveolar-capillary barrier. Compared to transwell-based platforms, the miniaturized scale allows to reduce the amount of reagents and cells towards medium and high throughput analysis. The alveolus-on-a-chip consisted of two PDMS micro-patterned layers sandwiching a PCL-Gel membrane that resemble the structural properties of the alveolar basement membrane. A supplementary layer was included to serve as a reservoir for the cell culture medium. A type I collagen hydrogel hosting fibroblasts (MRC-5) was loaded in the central well of the upper layer, designed to be completely open to atmosphere for recreating the ALI culture condition of alveolar epithelial cells. The microfluidic channels on the bottom layer were designed as serpentines to maximize the volume of cell culture medium in the basolateral chamber of the device. The design of serpentines, and the shape and the dimension of the device elements were optimized and characterized through optical microscopy and leakage tests.

To biologically validate the developed alveolus-on-a-chip, HEVC cells were seeded in the basolateral side of the PCL-Gel membrane, while A549 cells were cultured atop the MRC-5 - collagen hydrogel in the apical chamber of the device. To recreate the ALI condition, the culture medium on the apical chamber was removed after 3 days when the confluence of A549 cells was reached. Live/Dead and Immunostaining assays were performed to assess the cell viability, morphology, and expression of specific cell markers within the alveolus-on-a-chip. Finally, the formation of a functional barrier was investigated by immunostaining for zonula occludent (ZO-1), a multiprotein junctional complex, confirming the formation of tight junctions in the epithelial cells.

## 2 Materials and methods

### 2.1 Fabrication of PCL-Gel electrospun membrane

The PCL-Gel membranes were fabricated through electrospinning, as described previously (Giuntoli et al., 2021; Licciardello et al., 2023). Briefly, the polymeric (15% w/v) was obtained by mixing PCL (Mn = 80 kDa, Sigma Aldrich, Italy) and Gel (porcine skin, type A, Sigma Aldrich, Italy) in the ratio of 80:20 w/w in a mixture of formic acid (Sigma Aldrich. Italy) and acetic acid (Fisher scientific) (50:50 v/v). After 24 h of stirring, (3-Glycidyloxypropyl) trimethoxysilane (GPTMS, Sigma Aldrich, Italy) was added to the polymer solution in a concentration of 3.68% v/v to promote GL crosslinking (Tonda-Turo et al., 2013; Giuntoli et al., 2021). The solution was loaded into a 5 mL syringe with a 21-G needle and the electrospinning process was performed through the Novaspider instrument in vertical configuration (CIC nanoGUNE, Spain) exploiting the previously optimized process parameters (12 kV voltage, 500 μL h^-1^ flow rate and 12 cm distance). A plane collector was used to collect randomly oriented polymeric fibers.

### 2.2 Formulation of collagen hydrogel

The collagen hydrogel was formulated following a previously optimized protocol (Licciardello et al., 2023). Briefly, a 1 % wt./v. pre-hydrogel solution was prepared by dispersing type I collagen powders (bovine Achilles tendons, Blafar Ltd., Ireland) in a 0.5 M acid acetic solution in Eagle’s Minimum Essential Medium (EMEM, ATCC, Life Technologies, Italy) at 4°C. The solution was maintained at 4°C and after 12 h the solution pH was adjusted to 7.5 adding drop-by-drop of 10 M NaOH solution.

### 2.3 Design of the alveolus-on-a-chip

The device layers (60 mm × 20 mm) were designed through Rhinoceros^®^ CAD software (Robert McNeel and Associates). The master of each layer of the device was characterized by an external frame (1 mm thick x 2 mm high), designed to contain the casting of PDMS inside the mold.

**FIGURE 1:**
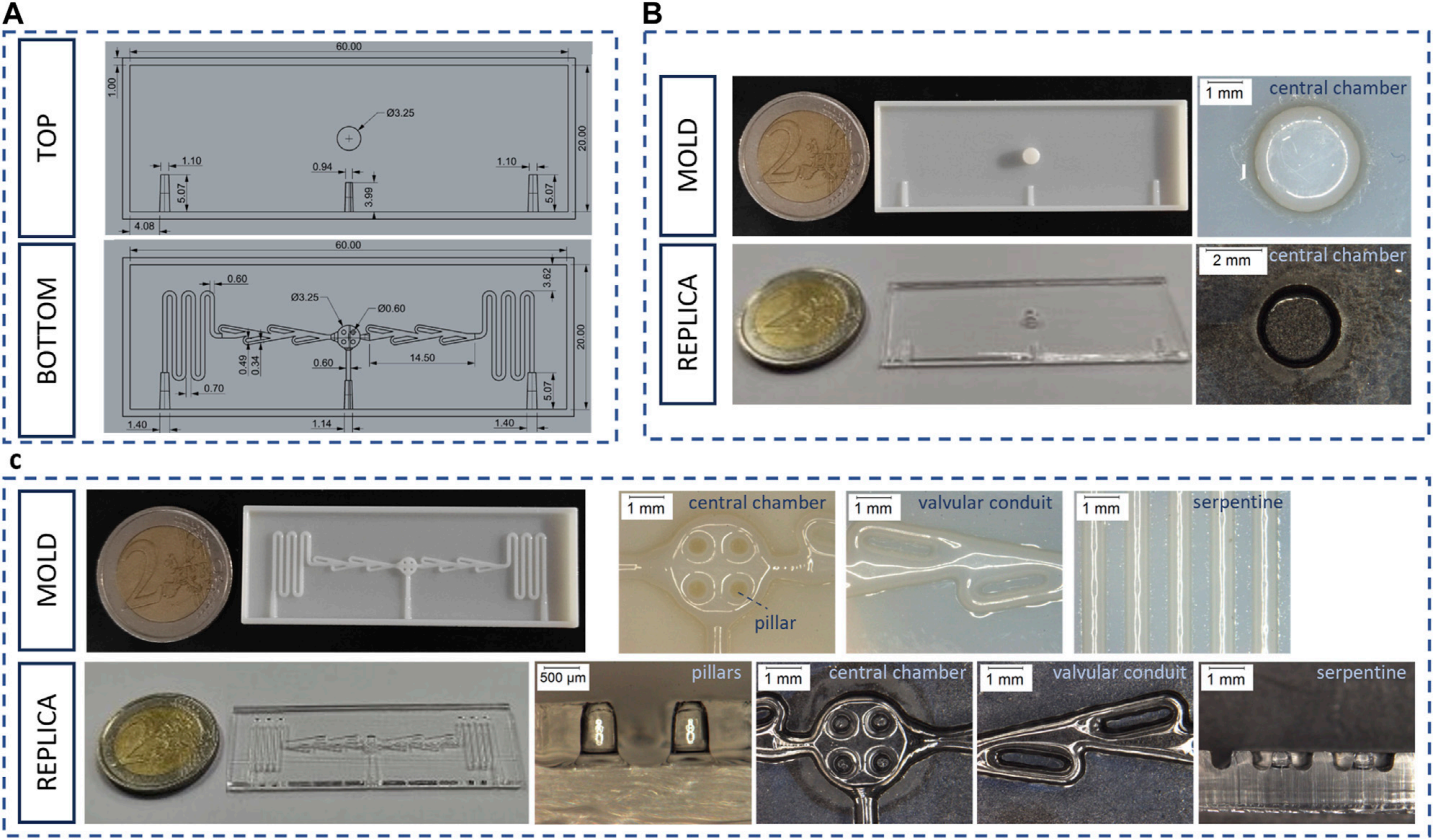
Bottom and top layer of the alveolus-on-a-chip. **(A)** CAD drawing of top layer and bottom layer **(B)** Photograph of the top layer mold and replica and optical microscope images of their characteristic elements. **(C)** Photograph of the bottom layer mold and replica and optical microscope images of their characteristic elements.

*BOTTOM LAYER.* The bottom layer (Figure 1A) has two sets of specular serpentines (600 µm width x 600 µm height) that can be accessed through two lateral inlets designed with a semi-truncated cone shape. The serpentine design was optimized to facilitate the flow of the cell medium from the inlet to the outlet. To this end, the microfluidic channels were connected to valvular conduits (Figure 1A), designed on the basis of Nikola Tesla’s original patent (Tesla, 1920). The valvular conduit shows convoluted shapes designed to create a preferential path for the medium flow. The valvular conduit connects to the central part of the master, designed to serve as culture chamber. This circular component (1 mm height) contains an array of four micropillars (600 µm in diameter) that aim to sustain the PCL-Gel electrospun membrane. Finally, a third smaller central inlet was designed for the seeding of endothelial cells, giving direct access to the culture chamber through a 600 μm × 600 μm channel. A custom mold was designed to fabricate a PDMS plug that allows to seal the central channel during the filling of the microfluidic channels (Supplementary Figure S1).

*TOP LAYER.* The top layer (Figure 1A) consists of a 3.25 mm central well completely open to atmosphere for recreating ALI condition. Three half-inlets with semi-truncated cone shapes were designed complementary with their corresponding halves in the bottom layer, in order to facilitate the filling process and prevent any leakage of medium from the inlet. Indeed, this configuration ensures that the 200 µL micropipette tips fit perfectly with the resulting conical shape inlet.

*RESERVOIR LAYER.* The final layer (Supplementary Figure S2) of the microfluidic device serves as a medium reservoir. This layer has an 8 mm central hole, concentric to the central well in the top layer, that allows to store about 100 µL of cell culture medium. Finally, a mold was designed to obtain a PDMS plug able to fit the concentric holes of top and reservoir layers. The plug allows to seal the upper layer facilitating the filling process of the microfluidic channel.

A schematic representation of the alveolus-on-a-chip assembly, comprising the different layers and the PCL-Gel electrospun membrane, was reported in Supplementary Figure S3.

### 2.4 Fabrication of the alveolus-on-a-chip

The molds for the alveolus-on-a-chip were fabricated through poly-jet 3D printing using OBJET30 Stratasys 3D printer. Briefly, each mold slice was obtained by extruding VeroWhite™ (Stratasys, United States, Minneapolis) photopolymer stacked layers and exposing the same while printing to UV radiation to induce crosslinking. The molds were placed in oven at 110°C overnight, then a rinse in acetone was performed to remove the remaining photo-initiators from the structures and promote recombination reactions between the photo-initiator and residual high molecular weight species in the photopolymer, more details about the procedure can be found in a previous work (Segantini et al., 2022).

The alveolus-on-a-chip layers were obtained through the replica molding method. Briefly, curing agent and PDMS pre-polymer (Sylgard 184 kit, VWR, Italy) were mixed with a ratio of 1:10 w/w. The solution was degassed in vacuum, obtaining a clear and transparent solution. The PDMS solution was cast on the molds and cured at 70°C for 90 min. Then, replicas were peeled off. PDMS layers were washed in ethanol by ultrasound for 30 s to remove dust and debris, that could interfere with the bonding process.

A 7 mm circular-shaped PCL-Gel membrane was punched with a biopsy punch and assembled between the top and bottom layers following surface activation using oxygen plasma treatment. In detail, the surface of the PCL-Gel membrane and channel sides of the top, bottom and reservoir PDMS layer were treated with oxygen plasma for 30 s with a power of 50 W using a low-pressure plasma system (Atto, Diener Electronic). After the treatment, the PCL-Gel membrane was placed on the central well of the top layer and sandwiched with the bottom layer. Finally, the reservoir layer was placed atop the top layer. The assembled device was incubated in a dry oven at 70°C for 10 min to promote the PDMS-PDMS binding (Marasso et al., 2017).

### 2.5 Characterization of the alveolus-on-a-chip

The PDMS layers of the device were characterized by comparing replica element dimensions with the CAD nominal dimensions using an optical microscope (Leica DVM2500, Leica Microsystems SRL, Italy, Milano). In particular, the bottom layers (n = 20) were sectioned to analyze the dimensions of pillars (height and diameter), cell chamber (diameter), and serpentines (diameter and distance). Furthermore, the central well of the top layer (n = 20) was analyzed.

Optical microscopy was also exploited to characterize the assembled device. To this end, the assembled alveolus-on-a-chip was sectioned with a sharp blade at the central well where the PCL-Gel membrane was housed. Moreover, in order to optimize and choose the right volume of hydrogel, the pre-hydrogel solution was prepared with Toluidine Blue and loaded into the central well of the top layer. In particular, hydrogel volumes of 5 μL and 10 µL were tested. After inducing the sol-gel transition at 37°C for 20 min, the devices were sectioned and analyzed using optical microscope.

A qualitative leakage test was performed to evaluate the sealing and the functionality of the alveolus-on-a-chip. A blue food dye solution in water in a ratio of 1:3 (v/v) was loaded into the inlet of the microfluidic channel in the bottom layer through a 200 µL micropipette. The non-quantitative test was performed under an optical digital microscope (Leica DVM2500), by observing the flow of blue food dye solution inside the microchannel of the device.

### 2.6 Cell culture

Human alveolar basal epithelial (A549) cells were obtained from ATCC and cultured in Roswell Park Memorial Institute 1,640 (RPMI, Gibco, Life Technologies, Italy) medium supplemented with 10% fetal bovine serum (FBS, Gibco, Life Technologies, Italy), 1% L-glutamine (L-Glu, Gibco, Life Technologies, Italy) and 1% penicillin/streptomycin (P/S, Gibco, Life Technologies, Italy). Human endothelial vein (HEVC) cells were obtained from Biology bank and Cell factory (IRCCS AOU San Martino - IST Italy)and cultured in Dulbecco’s Modified Eagle Medium (DMEM, high glucose, Gibco, Life Technologies, Italy) supplemented with 10% FBS, 2% L-Glu, 1% P/S. Human lung fibroblasts (MRC-5) were cultured in EMEM medium supplemented with 10% FBS and 1% P/S. Cells were maintained in a humidified incubator at 37°C and 5% CO_2_ on cell culture flasks.

### 2.7 Optimization of HEVC cell density on PCL-Gel membrane

The optimization of HEVC cell density on PCL-Gel membranes was performed by seeding cells in a transwell insert modified with the PCL-Gel membrane as we described previously (Licciardello et al., 2023). Briefly, a commercial transwell insert (CellQART^®^, SABEU GmbH and Co. KG, Germany) was modified by replacing the polyethylene terephthalate (PET) membrane with a square piece of PCL-Gel membrane on the external wall of the insert through a thin layer of PDMS. The modified transwell inserts were sterilized by incubating samples with 1% antibiotic-antimycotic (Gibco, Life Technologies, Italy) solution in PBS overnight. After washing twice with PBS, HEVC cells were seeded on the basolateral side of the PCL-Gel membrane at densities of 0.5 × 10^5^ and 1.5 × 10^5^ cells cm^-2^. The CellTiter-Blue^®^ viability assay (Promega) was performed at different time points (1, 3 and 7 days) to compare the viability of endothelial cells on the PCL-Gel membrane. After each time step, the medium in the basolateral chamber was removed and the samples were washed with PBS. According to the manufacturer’s protocol, CellTiter-Blue^®^ reagent was mixed with cell medium. The samples (n = 3 for each cell density) were incubated with 600 µL of medium containing CellTiter-Blue^®^ reagent and incubated at 37°C for 1 h. Then, 100 µL of solution was collected from the basolateral compartment and placed in a black 96-well plate. The fluorescence at 590 nm was recorded through SYNERGYTM HTX multimode plate reader (BioTeK, Winooski, VM, United States) to study the metabolic activity of cells.

In addition, LIVE/DEAD™ assay was performed to confirm the result of quantitative CellTiter-Blue^®^ viability assay. After washing samples with PBS, LIVE/DEAD™ reagents (Invitrogen, Life Technologies, Italy) were solubilized in PBS. Then, the basolateral chamber was filled with 600 µL of solution and the samples were maintained at room temperature for 40 min. Cells were imaged using Eclipse Ti2 Nikon confocal microscope.

### 2.8 Encapsulation of MRC-5 fibroblast into the collagen hydrogel

Before MRC-5 encapsulation, the collagen powders were exposed to UV light for 30 min in ice and the solution of acetic acid and EMEM was filtered using 0.22 μm filters. The pH of the pre-hydrogel solution was adjusted with a sterile 10 M NaOH solution. MRC-5 fibroblasts (80%–90% of confluency) were harvested and suspended in pre-hydrogel solution at 1.5 × 10 ^6^ cells ml^-1^, as we optimized in previous work (Licciardello et al., 2023).

### 2.9 Tri-culture in the alveolus-on-a-chip

The devices for cell tests were filled with 1% antibiotic-antimycotic solution and maintained at room temperature for 1 h. Afterward, the devices were rinsed twice with sterile phosphate buffer saline (PBS, Gibco, Life Technologies, Italy) and sterilized with UV light for 1 h.

HEVC cells were pelleted and resuspended in 10 μL of cell culture medium at the optimized cell density. HEVC cells were seeded in the basolateral side of the PCL-Gel membrane by pipetting the cell suspension through the central inlet. The device was flipped upside down and incubated at 37°C for 4 h. After this time, the device was flipped over and MRC-5 fibroblast-laden pre-hydrogel solution was poured into the apical central well. The device was incubated for 20 min to promote sol-gel transition. Then, A549 cells were suspended in 20 μL of MRC-5 medium and seeded atop the MRC-5-laden collagen hydrogel at 1.4 × 10^5^ cells cm^-2^ (Licciardello et al., 2023). The culture media were refreshed every day on both chambers of the device. The tri-culture was performed for 3 days under submerged (LLI) conditions. On day 3, the culture was switched to ALI by removing the cell culture medium from the apical chamber. In the LLI condition, MRC-5 growth medium was used for culturing cells in the apical chamber, while HVEC growth medium was used for the basolateral side. In the ALI condition, the apical chamber was exposed to air, and the basolateral chamber was rinsed with the HVEC growth medium. To avoid the dehydration of hydrogel, the microfluidic devices were placed in a Petri dish filled with 5 mL of sterile PBS. The tri-culture at ALI was carried out for 7 days (day 10 of culture in total). A schematic illustration of the arrangement of the different cell types within the alveolus-on-a-chip is reported in Supplementary Figure S4.

#### 2.9.1 Viability assay

The viability of cells inside the alveolus-on-a-chip was evaluated using LIVE/DEAD assay at different time steps (1, 3, 10 days of culture). Cells cultured within devices were incubated with LIVE/DEAD™ reagents (Invitrogen, Life Technologies, Italy), following the manufacturer’s instructions. Briefly, the basolateral and apical chambers of the devices were filled with 100 μL and 50 μL of LIVE/DEAD solution, respectively. After 40 min, each chamber of the device was rinsed once with PBS and cells were imaged using Eclipse Ti2 Nikon confocal microscope.

#### 2.9.2 Immunofluorescence staining

The immunofluorescence staining was performed to evaluate the morphology, the spatial distribution and the expression of specific markers of the tri-cultured cells in the alveolus-on-a-chip after 7 days at ALI. In particular, the culture medium was removed from the basolateral chamber and both chambers were rinsed with PBS. Cells were fixed by filling the device chambers with 4% paraformaldehyde in PBS (PFA, Fisher Scientific, Italy). After 40 min, PFA was removed, and chambers were rinsed twice with PBS. The samples were incubated with 0.2% v/v Triton X-100 (Sigma Aldrich, Italy) solution in PBS and maintained at room temperature for 10 min to permeabilize the cell membranes. After washing twice with PBS, the chambers were filled with a solution of 2% v/v of bovine serum albumin (BSA, Sigma Aldrich, Italy) in PBS for 1 h. Then, the samples were incubated with primary antibodies (Table 1) diluted in a solution of 1% v/v BSA and 0.1% v/v Tween 20 (Sigma Aldrich, Italy) in PBS at 4°C overnight. After rinsing twice with a solution of PBS with 1% v/v BSA and 0.1% v/v Tween 20, the chambers were filled with secondary antibodies (Table 1) diluted in the same solution and maintained at room temperature for 1 h. Finally, the samples were incubated with 4′,6-Diamidino-2-Phenylindole, Dihydrochloride (DAPI, Invitrogen, Life Technologies, Italy) solution (1:1,000 in PBS) for 5 min to stain the cell nuclei. After staining, the cells were imaged using Eclipse Ti2 Nikon confocal microscope. ImageJ software was used for image analysis. Fluorescence images of nuclei and cytoskeletons staining (×10 magnification) were used to evaluate HVEC adhesion within the device on 1 day. Specifically, the analysis was performed by comparing the count of cell nuclei with the administered cell count. Data are reported as mean ± standard deviation (*n* = 3).

**TABLE 1:** List of primary and secondary antibodies used for cell labeling in immunofluorescence staining.

Antibody	IgG subclass	Source	Dilution
Primary mouse anti- SP-C (H-8)	IgG2b	Santa Cruz Biotechnology	1:30
Primary mouse anti- ZO-1 (1A12)	IgG1	Invitrogen	1:100
Primary mouse anti- E-cadherin (HECD-1)	IgG_1_	Invitrogen	1:2000
Primary mouse anti- AQP-5 (D-7)	IgG1	Santa Cruz Biotechnology	1:50
Primary rabbit anti- vimentin (SP20)	IgG	Invitrogen	1:1,000
Secondary Cyan5 goat anti-mouse	IgG (H + L)	Invitrogen	1:1,000
Secondary Alexa Fluor 488 goat anti-mouse	IgG (H + L)	Invitrogen	1:1,500
Secondary Alexa Fluor 555 goat anti-rabbit	IgG (H + L)	Invitrogen	1:1,000

#### 2.9.3 Evaluation of LPS-induced inflammation in the alveolus-on-a-chip

Lipopolysaccharide treatment was performed in the tri-culture model after 7 days of culture at ALI. Briefly, the cell medium was removed from the basolateral compartment of the chip, and samples were rinsed with sterile PBS. Then, the apical compartment was treated with 4 μg/mL lipopolysaccharide (LPS, Sigma Aldrich) in MRC-5 culture medium for 2 days, while the basolateral chamber was filled with HVEC culture medium. The effect of LPS treatment was investigated by immunostaining for ZO-1 marker in A549 cells.

### 2.10 Statistical analysis

Data were analyzed with two-way ANOVA using GraphPad Prism 9.3.1 software (San Diego, CA, United States).

## 3 Results

### 3.1 Characterization of the alveolus-on-a-chip

#### 3.1.1 Evaluation of molds and replicas fidelity for the top and bottom layer

The molds and PDMS replicas of the top and bottom layers (Figure 1) of the alveolus-on-a-chip were characterized by analyzing their main geometric features through optical microscopy. Table 2 shows the comparison between the experimental measures of molds and replicas and the CAD theoretical dimensions. All the elements of the molds maintained the desired geometry after 3D printing process (Figures 1B, C). In particular, the top layer mold consisted of a central well of 3,003 ± 1 µm in diameter while the bottom layer presented a central chamber of 3,048 ± 4 μm, with 4 pillars of 544 ± 54 µm in diameter.

**TABLE 2:** Nominal and experimental dimension of the top and bottom layer of the alveolus-on-a-chip.

LAYER		CAD (µm)	MOLD (µm)	Replica (µm)
**TOP**	Central well	3,250	3,003 ± 1	3,272 ± 183
**BOTTOM**	Central chamber	3,250	3,048 ± 4	3,183 ± 64
	Valvular conduit diameter	340	422 ± 2	463 ± 23
	Serpentine diameter	600	568 ± 1	618 ± 32
	Serpentine distance	700	524 ± 1	522 ± 43
	Pillar diameter	600	544 ± 54	526 ± 33
	Pillar height	900	—	830 ± 98

In comparison, the top layer replica showed a central well of 3,272 ± 183 µm in diameter. The bottom layer consisted of a central chamber of 3,183 ± 64 µm in diameter and two serpentines with valvular conduits of 463 ± 23 µm in diameter. The pillars in the bottom chamber showed a diameter of 526 ± 33 µm and a height of 830 ± 98 µm. In general, the experimental dimensions were comparable with the theoretical values, confirming the success of replica molding process.

#### 3.1.2 Characterization of the assembled device

**FIGURE 2:**
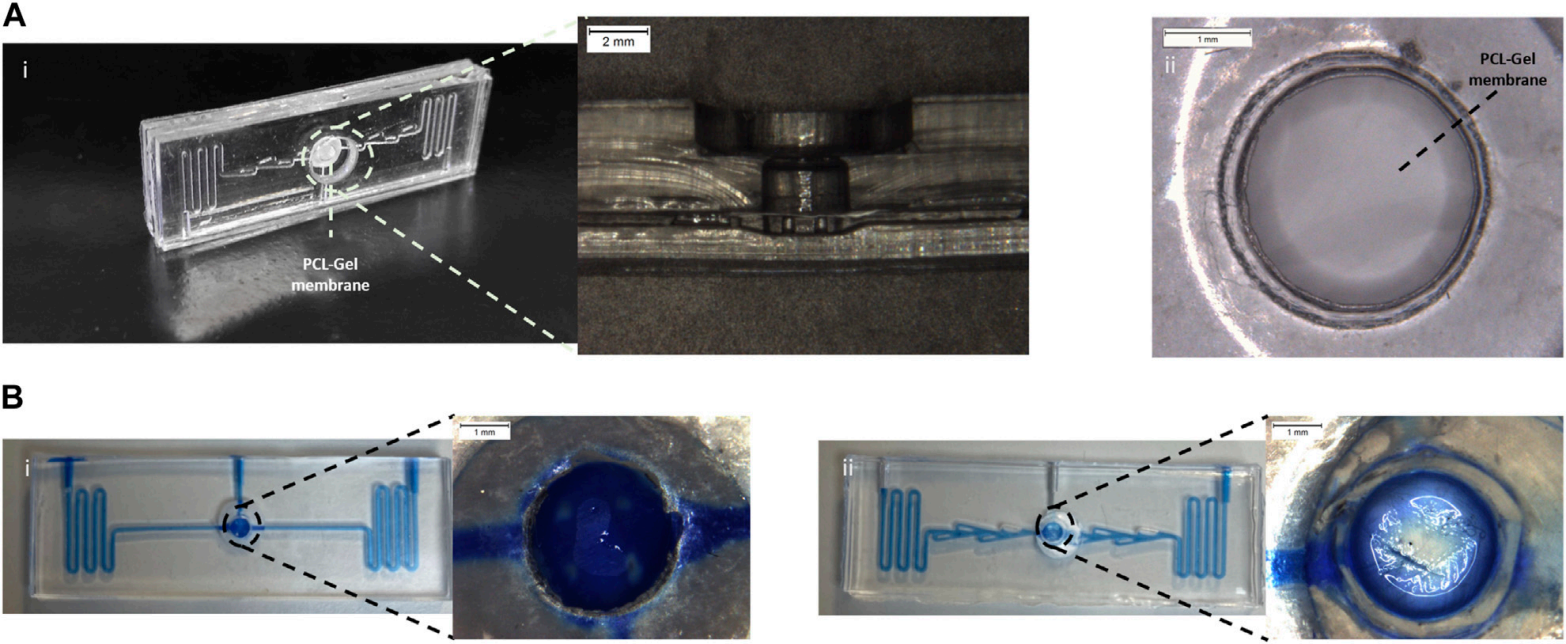
Assembled alveolus-on-a-chip. **(A)** Photograph and an optical microscope image of a section of the assembled device **(i)** and PCL-Gel membrane **(ii)**. **(B)** Static leakage tests performed in the device layout without **(i)** and with **(ii)** valves.

The assembled device was obtained through plasma oxygen treatment. The optical microscopy image of the assembled device (Figure 2A) confirms that the pillars were not compressed during assembling and the PCL-Gel electrospun membrane fitted perfectly inside the device being correctly supported by pillars without deformations.

A static leakage test (Supplementary Video S2) was used to validate the bonding process. No leaking or layer separation was observed demonstrating good sealing of the microfluidic device mediated by the plasma oxygen process.

In addition, the leakage test was exploited to evaluate the functionality of the valvular conduit. To this end, a device without valves was fabricated and tested. As shown in Figure 2B, in the device layout without valves (Figure 2Bi) the liquid level rises in the top layer submerging the PCL-Gel upper layer. The introduction of valves (Figure 2Bii) in the microchannel allowed to create a preferential path for the fluid flow limiting the flow of the blue food dye in the central microchannel without invading the apical chamber.

Moreover, a collagen pre-hydrogel solution was prepared using a mixture of medium and Toluidine Blue and loaded into the central well of the device to optimize the volume of hydrogel to be used. As shown in Supplementary Figure S5 a volume of 5 ul did not allow to obtain a hydrogel layer that homogenously covered the apical surface of the PCL-Gel membrane. Conversely, a more homogeneous layer was observed for 10 ul of volume. Thus, 10 ul was selected as optimal volume of hydrogel and used for the biological validation of the device.

### 3.2 *In vitro* cell tests

#### 3.2.1 Optimization of HVEC cell density on PCL-Gel membrane

**FIGURE 3:**
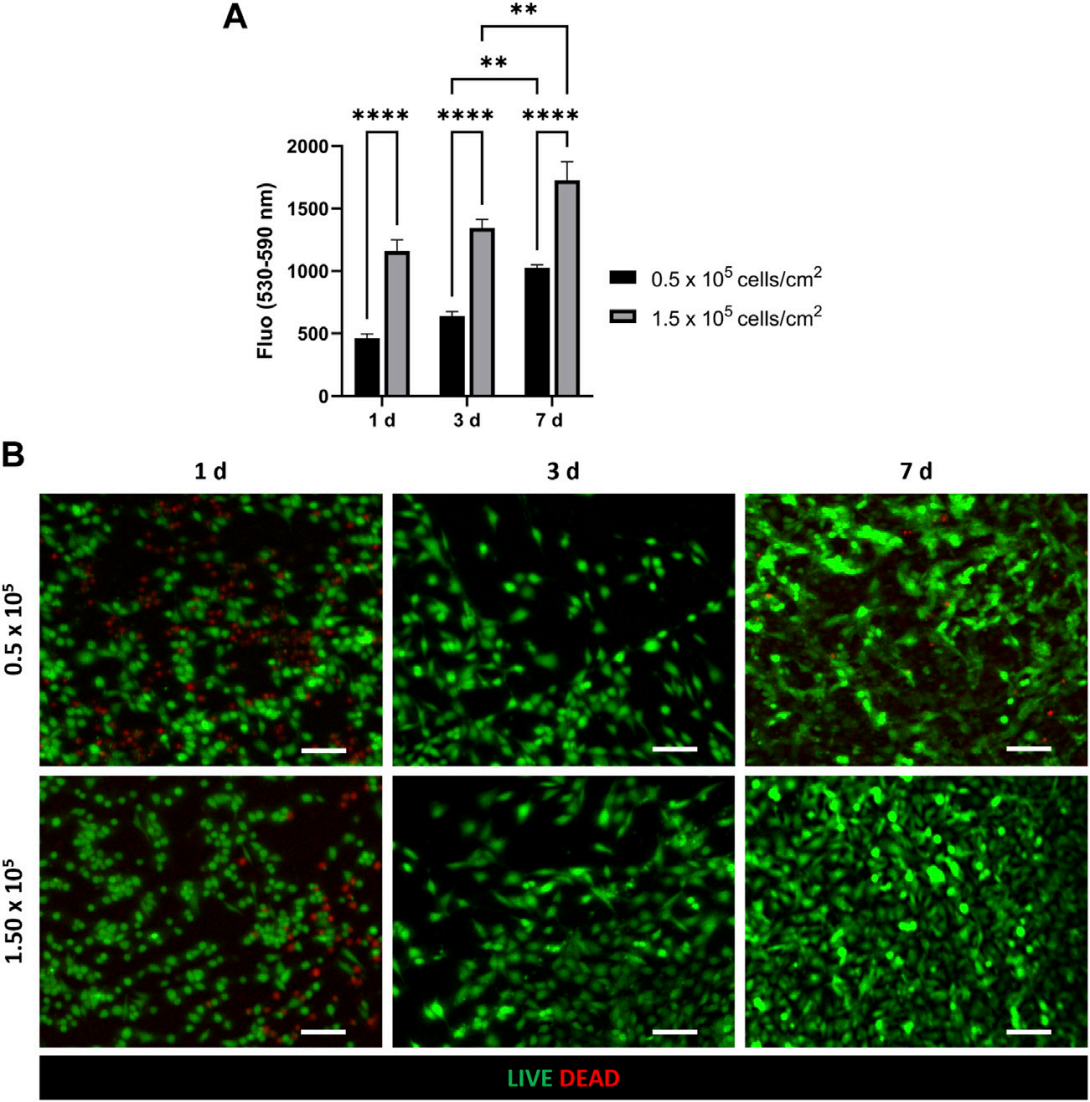
Optimization of HVEC cell density on PCL-Gel membrane. **(A)** Quantitative evaluation of HVEC cell viability with CellTiter-Blue^®^ assay. Statistical difference (**p < 0.01; ****p < 0.0001). **(B)** Representative LIVE/DEAD images (*n* = 3) of HVEC seeded at different cell densities (5 × 10^5^ and 5 × 10^5^) at 1 day, 3 days and 7 days after seeding. ×10 magnification images (scale bar = 100 μm).

CellTiter-Blue^®^ viability assay and LIVE/DEAD assay were used to evaluate HVEC viability and proliferation on PCL-Gel membrane and assess an appropriate value of cell density to achieve cell confluency on the substrate in the culture period (Figure 3). In particular, CellTiter blue viability assay (Figure 3A) showed an increase in cell viability between day 3 and day 7 for both the tested densities. However, cells seeded at 1.5 × 10^5^ cells/cm^2^ density resulted more metabolically active and viable compared to cells seeded at a lower cell density (0.5 × 10^5^ cells/cm^2^), demonstrating as the higher density resulted in optimal viability and proliferation of HVEC cells on PCL-Gel membranes. This trend was confirmed by LIVE/DEAD™ images. Indeed, as shown in Figure 3B, HVEC cells cultured at 1.5 × 10^5^ cells/cm^2^ density were able to better grow during the culture period, creating a compact cellular monolayer that homogeneously covered the entire surface of PCL-Gel membrane after 7 days in culture.

#### 3.2.2 Cell tri-culture in the microfluidic device

**FIGURE 4:**
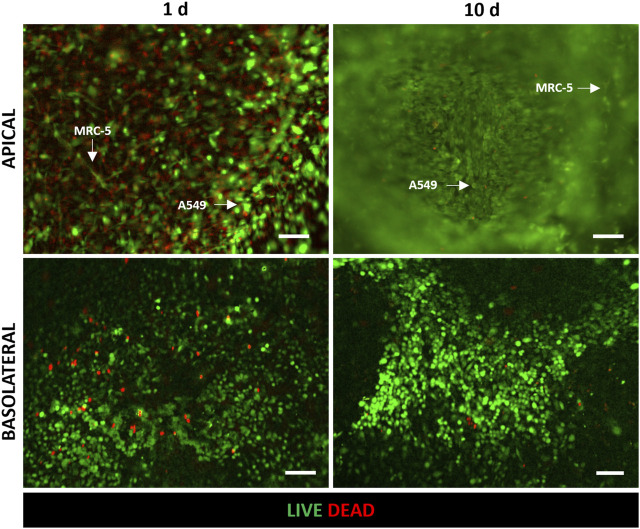
Evaluation of cell viability after 1 day and 10 days (7 days at ALI) of culture in the alveolus-on-a-chip. Representative fluorescence images (*n* = 3) of LIVE/DEAD assay in HVEC (in the basolateral chamber), A549 and MRC5 (in the apical chamber) tri-culture. Live and dead cells exhibited green and red fluorescence, respectively. ×10 magnification images (scale bar = 100 μm).

The tri-culture model was implemented by seeding HVEC cells in the basolateral chamber of the alveolus-on-a-chip. MRC-5-laden hydrogel was poured on the apical central well and the device was incubated at 37°C to achieve collagen fibrillogenesis. Then A549 cells were seeded on the top of the hydrogel. The ALI condition was established after 3 days by removing the medium on the apical chamber and leaving the A549 cells exposed to air. The tri-culture at ALI was performed for additional 7 days and then the behavior of tri-cultured cells was evaluated in terms of cell viability, cell morphology and expression of specific markers. Figure 4 shows the LIVE/DEAD™ images of tri-cultured cells. In particular, the images confirmed the presence of HVEC cells in the basolateral chamber and both A549 and MRC-5 cells in the apical chamber (the typical elongated and polygonal morphologies of fibroblasts and epithelial cells can be observed, respectively). Interestingly, the number of dead cells was significant at day 1 but it remarkably decreased at day 10 (day 7 at ALI). Thus, LIVE/DEAD™ images (Figure 4) demonstrated that the tri-cultured cells proliferated and remained viable for up to 7 days at ALI.

HVEC adhesion within the device was further evaluated by fluorescence imaging to assess the reproducibility of the seeding process through the central inlet and its impact on cell loss. Supplementary Figure S6 reports representative fluorescence images of HVEC nuclei and cytoskeletons at different magnifications (4x and 10x) after 1 day from seeding. The images show a homogenous cell distribution on the PCL-Gel membrane, especially in the central seeding area. According to the count of cell nuclei, the percentage of cells attached to the central seeding area between the pillars is 33% ± 7% of the administered cell count (n = 3). The uniform arrangement of cells and the low variation in the nuclei count among the samples confirmed the efficacy of the seeding process, despite its criticality.

**FIGURE 5:**
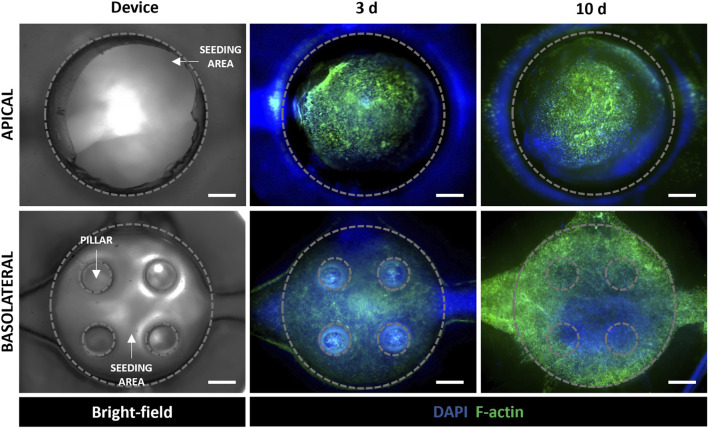
Evaluation of cell behavior in the alveolus-on-a-chip. Bright-field image of the apical and basolateral chambers of the chip (left). Representative fluorescence images (*n* = 3) of cytoskeleton staining in a549 and MRC5 (in the apical chamber), and HVEC (in the basolateral chamber) tri-culture after 3 days and 10 days (7 days at ALI). 4 x magnification images (scale bar = 500 μm).

**FIGURE 6:**
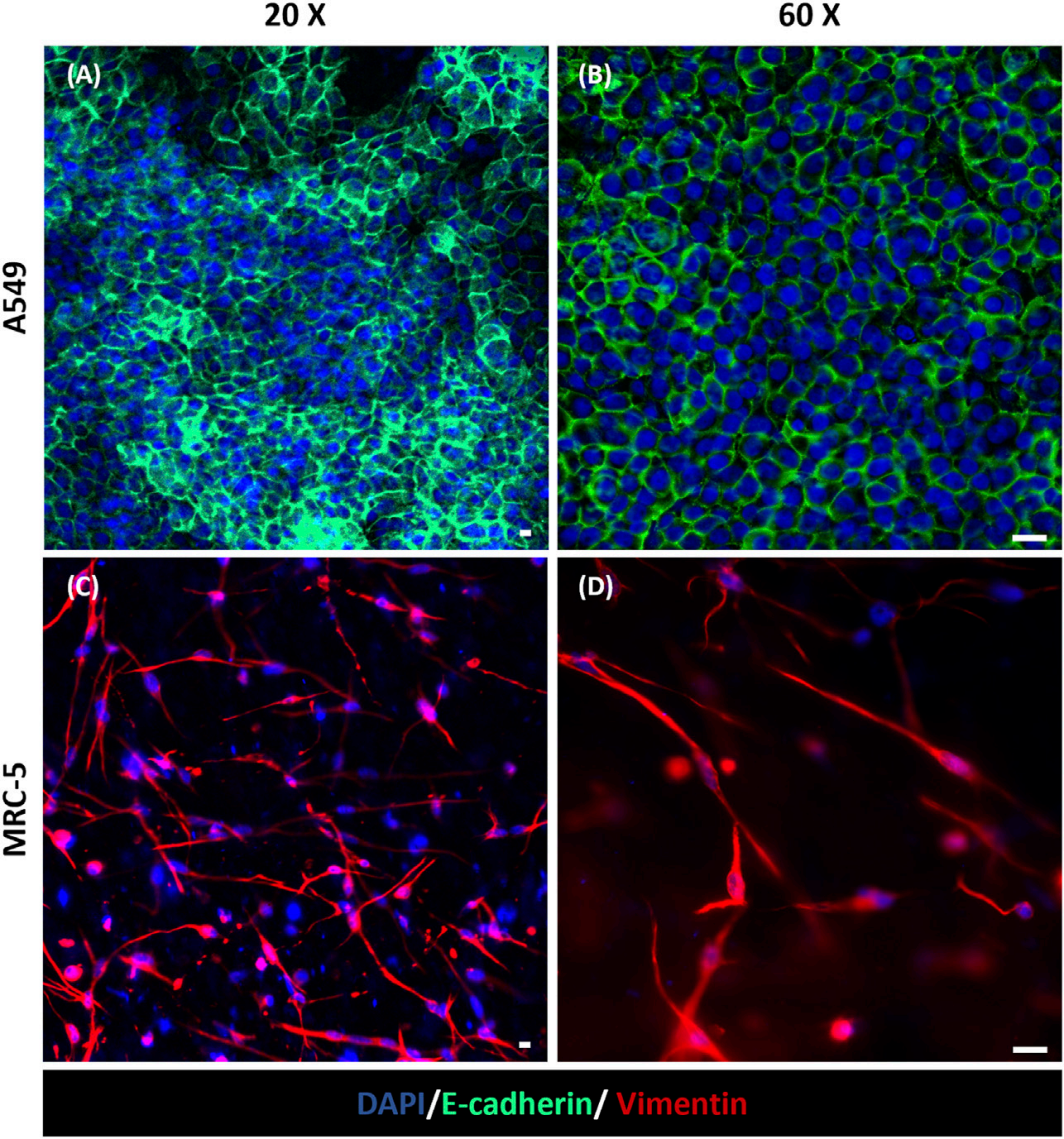
Immunofluorescence staining for E-cadherin and Vimentin in the apical chamber of the alveolus-on-a-chip. Expression of E-cadherin (green) in A549 cells **(A, B)** and Vimentin (red) in MRC-5 fibroblasts **(C, D)** after 10 days (7 days at ALI) from the same optical field imaged at a different z stack position. **(A, C)** ×20 magnification images (scale bar = 10 μm) and **(B, D)** ×60 magnification images (scale bar = 20 μm) (*n* = 3).

The fluorescence staining of cell nuclei and cytoskeletons was used to evaluate cell morphology and distribution within the device. In particular, as shown in Figure 5, A549 cells in the apical side of the device reached confluency and formed a uniform cell layer on the top of the MRC-5-laden collagen hydrogel after 3 days of tri-culture under LLI. Then, this time step was chosen to implement the ALI condition because epithelial cells have to form a confluent cell layer before establishing ALI (Huh et al., 2010; Ren et al., 2016). Moreover, confocal images at 10 days (7 days at ALI) show confluent layers of A549 and HVEC cell layers that presented their characteristic polygonal morphology confirming that cells were functionally active within the device (Figure 5 and Supplementary Figure S7). However, in the images at low magnification (Figure 5) is difficult to recognize MRC-5 fibroblasts that are embedded in the collagen hydrogel and then located in the inner portion of the apical chamber of the device. Immunofluorescence images at higher magnification (20x and 60x) were captured to evaluate the presence of MRC-5 fibroblasts in the tri-culture system and to specifically distinguish MRC-5 and A549 cells (Figure 6). In particular, confocal images obtained from z-stack acquisition (Supplementary Video S1, Supplementary Figure S8) demonstrated the presence of MRC-5 fibroblasts (within collagen hydrogel) and A549 (atop collagen hydrogel) that expressed vimentin and E-cadherin markers, respectively. In particular, MRC-5 displayed characteristic spindle-shaped morphology and appeared homogeneously dispersed within the hydrogel. In addition, images of the basolateral chamber were captured in the same optical field, assessing the presence of a confluent monolayer of HVEC cells on the opposite side of the PCL-Gel membrane (Supplementary Figure S9).

**FIGURE 7:**
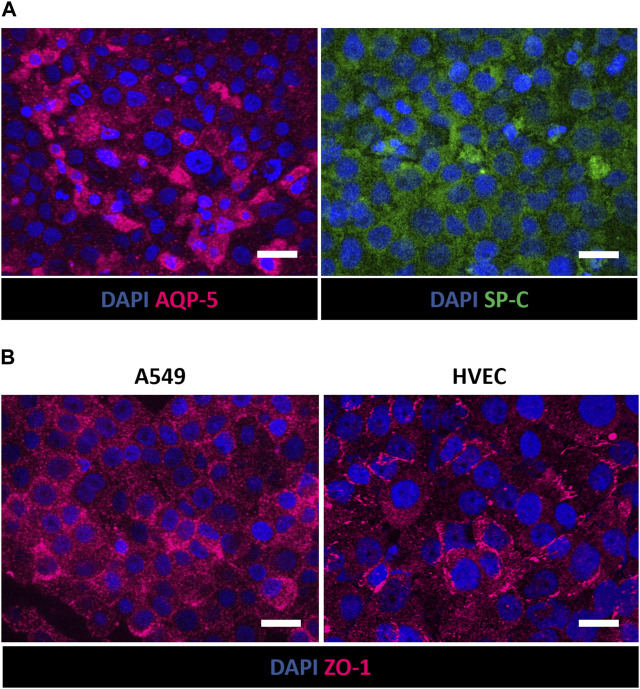
Immunofluorescence staining of A549 and HVEC cells in the apical and basolateral chamber of the alveolus-on-a-chip. Expression of AQP-5 (magenta) and SP-C (green) in A549 cells **(A)** and ZO-1 (magenta) in A549 (left) and HVEC (right) **(B)** after 10 days (7 days at ALI). ×60 magnification images (scale bar = 20 μm) (*n* = 3).

Moreover, immunofluorescence staining for AQP-5 and SP-C in A549 cells demonstrated the expression of type I and type II alveolar epithelial markers, respectively, after 7 days at ALI (Figure 7A). The recreation inside the alveolus-on-a-chip of epithelial and endothelial tight and functional barriers was also demonstrated by assessing the expression of tight junctions ZO-1 in A549 and HVEC cells. As shown in Figure 7B, both cell lines were positively stained for markers showing a more diffuse expression of junctional markers at the cell border.

Lastly, LPS treatment was performed in the alveolus-on-a-chip to evaluate the potential of the tri-culture system in modeling alveolar inflammation. Specifically, cell response to the inflammatory stimulus was investigated by analyzing changes in the expression of tight junctions ZO-1 in A549 cells after 2 days of treatment. As shown in Supplementary Figure S10, a lower fluorescence was observed for LPS-treated samples, while untreated A549 displayed a higher and more diffuse expression of ZO-1. These results suggested that LPS-induced inflammation may compromise the integrity of the epithelial barrier in the model, due to damage of tight junctions between cells (Kim et al., 2023).

## 4 Discussion

The alveolar-capillary barrier is the site of gaseous exchange between the air space and pulmonary capillary (Leiby et al., 2020). Several acute and chronic lung diseases result in remodeling of the alveolar region and damages of the alveolar-capillary barrier (Evans and Lee, 2020). Despite different therapies are used to treat lung disease, many patients present a low life expectancy (Barnes et al., 2015). Thus, the development of new therapies is still urgently needed to reduce the incidence and deleterious effects at the alveolar level of lung diseases.

Over the past decade, only about 3% of new drugs achieved clinical approval due to the poor understanding of disease pathogenesis and the lack of effective preclinical models that rapidly predict the efficacy of new therapeutic approaches in humans (Ioannidis et al., 2018; Soriano et al., 2021). Indeed, in addition to ethical concerns, the use of preclinical animal models has revealed a lack in the ability to replicate human disease and correctly predict the effect and the response to therapy (Greek and Kramer, 2019). Moreover, the traditional 2D *in vitro* models fail to replicate the complex multicellular interaction and three-dimensional (3D) architecture of lung tissue as well as the physiological microenvironment of healthy and pathological lung (Duval et al., 2017; Kumar et al., 2018; Öhlinger et al., 2019; Bassi et al., 2021). For these reasons, more effective preclinical models are required to improve the success of drug development (Low et al., 2021). The implementation of innovative *in vitro* strategies is a suitable alternative that allows to mimic the healthy and pathologic state of the human lung. In the last decade, the advent of microfabrication techniques has given the possibility to implement downscaled engineered systems, obtaining microfluidic devices for medium and high-throughput analysis. In particular, lung-on-a-chips have been proposed as robust lung *in vitro* models thanks to their ability to replicate the complex architecture, both cell-cell and cell-ECM interaction and physiological cues within healthy and pathological lungs. Polydimethylsiloxane (PDMS), an elastomeric silicon-based polymer, is widely used to fabricate microchip devices, thanks to its biocompatibility, thermal stability, chemical inertia and gas permeability (Miranda et al., 2022). Soft-lithography techniques are exploited for the fabrication of lung-on-a-chips, allowing to reproduce the desired pattern with a high resolution and complex design (Cho et al., 2023; Wang et al., 2023). Indeed, lung-on-a-chip devices are designed to possess continuously perfused microchannels, ranging in size from tens to hundreds of μm (Low et al., 2021), occupied by living cells (Hassell et al., 2017). Typically, lung-on-chip platforms consist of a two-chamber system, in which the cell culture substrate is accommodated between the apical and basolateral chambers. Although these devices represent a crucial progress in the field of *in vitro* models, they still fail to emulate the behavior of the physiological ECM. Indeed, typically, a porous membrane made of PDMS is used as cell culture substrate which differs from the native lung ECM in composition, topography and stiffness (Zhou et al., 2018). Moreover, the PDMS hydrophobicity led to a high capacity to adsorb and absorb small molecules (Boxshall et al., 2006; van Meer et al., 2017), limiting the presence of bioactive molecules and growth factors in the microenvironment at a physiological concentration. Thus, an important challenge is the replacement of PDMS membranes with advanced ECM-like structures made of biomaterials that replicate the properties and functions of lung tissue. To this end, in this work, we propose the implementation of a biomimetic microfluidic platform that resembles the multilayered architecture of the alveolar-capillary barrier and the composition of alveolar ECM, physiologically composed by a thin basement membrane and a dense fibrous interstitial space. The alveolus-on-a-chip was designed to include a PCL-Gel electrospun membrane between two PDMS micro-patterned layers molded from two masters obtained through poly-jet 3D printing. As we recently reported (Licciardello et al., 2023), the PCL-Gel nanofibrous membrane reproduces the composition and morphology of the alveolar basement membrane. The top layer of the device presented a central well completely open to the atmosphere to recreate the ALI culture condition of alveolar epithelial cells and allows to host a collagen hydrogel encapsulating lung fibroblasts to mimic the fibrous interstitial space. The bottom layer had a central microchannel with two sets of serpentines designed to maximize the volume of cell medium that can be stored in the microchannel ensuring the cell survival. A valvular conduit was designed on the basis of Tesla’s original patent (Tesla, 1920) and introduced within the microfluidic channel. In previous works, Tesla’s valves were studied for microfluidic applications and used in microfluidic mixer networks for the creation of gradients to obtain convective mixing of flows (Hong et al., 2004; Habhab et al., 2016). Here, the valvular conduit was designed to steer the flow of cell medium from the inlet to the outlet, counteracting the ascent of the medium into the apical chamber through the membrane, thanks to its direction-dependent fluidic resistance given by the convoluted shapes that create a preferential path for culture medium flow (Nguyen et al., 2021). This feature is necessary for the long-term maintenance of the ALI condition. Moreover, in the bottom central chamber, an array of 4 micropillars was fabricated to sustain the cell culture membrane.

The 3D printed molds and the PDMS replicas of the top and bottom layers were analyzed using optical microscopy to evaluate the fidelity to the nominal dimensions (Table 2). The slight difference between the solid elements of the molds and the CAD model quote could be ascribed to the small delay between the liquid resin layer deposition and the UV curing process.

In general, the geometry of the PDMS replicas showed a good resolution. However, the dimensions of device elements which appeared more deviate from the nominal value were the pillar diameter, the serpentine distance, and the valvular conduit diameter. The leakage test (Figure 2B) demonstrated that the blue food dies flowed through microfluidic channels without fluidic resistance demonstrating as the difference between nominal and experimental value of serpentine distance and valvular conduit diameter did not affect the fluidic properties of the device. The pillars are fundamental elements designed to sustain the cell culture substrate. Indeed, as shown in Supplementary Figure S11, the electrospun membrane bends and curves due to the absence of these support structures in the culture chamber of the bottom layer. Conversely, when the pillars were introduced in the bottom chamber, the PCL-Gel membrane was correctly supported by pillars without deformations. The pillar height is the characteristic parameter that greatly affects the ability of pillars to sustain the cell culture substrate while pillar diameter does not affect the fluidic properties of the device nor the membrane stability (Marasso et al., 2017). Then, the difference between nominal and theoretical values of pillar diameters should not affect the function of these structures.

The oxygen plasma treatment was exploited to bond the PDMS layers, obtaining the final assembled devices (Figure 2A) having the PCL-Gel membrane sealed between the two PDMS layers. Indeed, exposing PDMS to the plasma oxygen, the methyl groups (CH_3_) on the PDMS surface were replaced by the hydrophilic silanol groups (Si-OH) leading to the increase of material hydrophilicity (Mata et al., 2005). Thus, the intimate contact between the activated PDMS surfaces promoted the stability of the device assembling thanks to the formation of irreversible Si-O-Si bonds (Wu et al., 2013). The leakage test (Figure 2B) confirmed the success of the plasma oxygen process demonstrating the good sealing of the assembled device. Indeed, the presence of valvular conduit (Figure 2Bii) in the microchannel ensured a lower tendency to liquid level rising above the PCL-Gel membrane in the apical chamber compared to the device layout without valves (Figure 2Bi).

The biological validation of the device was carried out by implementing the tri-culture of endothelial (HVEC), fibroblast (MRC-5) and epithelial (A549) cells within the alveolus-on-a-chip. In particular, HVEC cells were seeded in the basolateral chamber of the device. MRC-5 fibroblasts were embedded in a collagen hydrogel to reproduce the alveolar interstitial space. The MRC-5-laden hydrogel was loaded in the apical central well and then A549 cells were seeded on the top to recreate the alveolar epithelium. The tri-culture was maintained 3 days under submerged conditions and the ALI culture condition was recreated by removing the medium in the apical compartment and leaving epithelial cells completely exposed to air for additional 7 days. LIVE/DEAD assay revealed the good adhesion and the remarkable viability of cells demonstrating as the chip design allowed for functional cell seeding and maintenance of cell viability up to 7 days at ALI (Figure 4). The high number of dead cells on day 1 could be attributed to the seeding process that could be slightly aggressive for cells (Sgarminato et al., 2022). Indeed, the fibroblast encapsulation process could cause physical stress for cells and some cells may die in that process. Moreover, while the open central well in the upper layer allows direct access for the cell seeding, the HVEC cell suspension has to flow through the central PDMS inlet to reach the bottom chamber resulting in a more critical seeding process which could affect cell viability within the first hours after seeding. Figure 5 provided a schematic representation of cell distribution at 3 days of culture and 7 days at ALI, confirming the presence of compact endothelial and epithelial monolayers in the apical and basolateral chambers, respectively. By comparing cell behavior before and after ALI establishment, no evident differences in cell attachment and morphology were observed. Therefore, cells seeded atop the membranes did not experience a reduction in cell viability when exposed to air confirming the diffusion of a sufficient amount of metabolites from the bottom layer. This is a key point of the here proposed device considering the small dimensions of the device chambers as well as the reduced volumes of culture medium they can accommodate. The design of microchannels and basolateral chamber enabled the endothelial cell adhesion and proliferation on the PCL-Gel membrane. In particular, the creation of serpentine hindered the medium evaporation and reduced the shear stress on seeded cells. HVEC cells in the basolateral chamber covered the entire seeding area showing an active proliferation within the ALI culture period (Figure 5). Interestingly, a reduction of the hydrogel surface was observed by comparing it with the diameter of the apical central well. This tendency was particularly evident with the increase of the culture period, indicating the contraction of MRC-5-laden hydrogel in response to fibroblast proliferation and interaction with binding sites of collagen hydrogel (Smithmyer et al., 2014; Camasão et al., 2019). Indeed, several studies highlighted the role of collagen matrices in influencing lung fibroblast behavior (Hackett et al., 2022; Phogat et al., 2023), mimicking the structural and functional features of lung tissue. The developed collagen hydrogel formulation was previously demonstrated to successfully support the growth of MRC5 fibroblasts and show mechanical properties comparable to hydrogels derived from lung ECM (Pouliot et al., 2016; Licciardello et al., 2023).

Moreover, the presence of the hydrogel was shown to support A549 adhesion and viability at ALI, providing an optimal biomimetic environment for fibroblast and epithelial cell co-culture. More in detail, the immunofluorescence analysis revealed that cultured cells in the apical side of the device expressed phenotype-specific markers. Indeed, Figures 6A, B shows the presence of E-cadherin positive-stained A549 cells on the surface of collagen hydrogel, confirming the expression of characteristic epithelial adherent junctions in the cell monolayer (Morris et al., 2014; Zamprogno et al., 2021; Zhang et al., 2022) that play a pivotal role in maintaining cell-cell adhesion beyond as well as tissue integrity (Rubtsova et al., 2022). Moreover, vimentin was used to identify encapsulated MRC-5 fibroblasts being an effective marker for fibroblasts (Goodpaster et al., 2008; Ostrowska-Podhorodecka et al., 2022). As shown in Figures 6C, D, MRC-5 fibroblasts appeared uniformly widespread within the collagen matrix. The recreation of ALI culture is fundamental to simulate the alveolar physiological environment. Despite the integration of ECM-like substrate within alveolus-on-a-chip devices was previously reported in literature (Higuita-Castro et al., 2017; Yang et al., 2018; Zamprogno et al., 2021), the replication of air exposure in a device that reproduces the interaction between the alveolar epithelium, alveolar interstitial space and the pulmonary endothelium, was not previously achieved. Here, the innovative design of the microfluidic device allows the air exposure of the apical compartment, supporting the establishment of ALI without affecting hydrogel hydration and then cell viability. Furthermore, the simulation of the exposure to air experienced by epithelial cells in a physiological environment promoted the expression of type I and type II alveolar epithelial cell markers AQP-5 and SP-C, respectively (Figure 7A) (Baptista et al., 2022), and then the differentiation of A549 cells into alveolar epithelial cells, in accordance with previously obtained results (Wu et al., 2018; Öhlinger et al., 2019; Licciardello et al., 2023). Lastly, the expression of ZO-1 in A549 and HVEC cells (Figure 7B) demonstrates the formation of a functional alveolar-capillary barrier (Pasman et al., 2021; Zamprogno et al., 2021; Jain et al., 2023) within the alveolus-on-a-chip.

The obtained results show the potential of this platform in the modeling of the alveolar-capillary barrier. Indeed, the establishment of a tri-culture system provides the possibility of investigating the interaction between endothelium, stroma, and epithelium with more physiological relevance compared to existing co-culture systems. Specifically, the model has been designed to reproduce both the cellular and extracellular compartments in terms of composition and structure, aiming to create a biomimetic 3D environment for cell culture. Indeed, several studies reported the increased functionality of 3D culture systems compared to 2D counterparts (Kang et al., 2021; Licciardello et al., 2023; Phogat et al., 2023). For example, Kang et al. (2021) demonstrated the efficacy of 3D structured models in better recapitulating the features of the alveolar barrier compared to 2D ones, in terms of barrier integrity and expression of alveolar-specific genes. Here, the presence of the interstitial layer significantly enhances the biomimicry of the alveolus-on-a-chip model compared to other systems, mostly focused on the reproduction of the epithelial and endothelial barrier. Indeed, previous studies underlined the importance of the interaction between the stroma and epithelium in the modeling of inflammatory-related alveolar disorders or cancer (He et al., 2017; Koukourakis et al., 2017; Movia et al., 2019; Doryab et al., 2022). For example, Doryab et al. (2022) developed a human-based triple coculture fibrosis model, including epithelial and endothelial cell lines and primary pathological fibroblasts in a transwell-like system. Here we use normal fibroblasts embedded into a collagen hydrogel to better recreate the 3D distribution of cells inside the alveoli creating a healthy model to be used in the study of early stages of pathology development. In addition, the scale size of the here developed platform allows to reduce the amount of reagents and cells towards medium/high throughput analysis. Moreover, the use of alveolus-on-a-chip for modeling pathological conditions was preliminarily assessed by investigating the effect of LPS-induced inflammation on epithelial cells, suggesting the applicability of this system for the study of inflammatory-related alveolar disorders.

## 5 Conclusion

A new design to create an alveolus-on-a-chip was proposed in this work. The geometry of the bottom layer and the top layer permits the development of a tri-culture 3D system, comprising the most representative cell phenotypes of the alveolar-capillary barrier as well as the establishment of an air-liquid interface condition, fostering the development of a more physiologically relevant environment. Furthermore, the inclusion of a fibrous interstitial space between the epithelium and the endothelium was included in a microfluidic device for the first time, allowing for the replication of the thick portion of the of the alveolar-capillary barrier. The here developed platform can become a promising tool to study *in vitro* inflammatory-related pathologies (e.g., fibrosis and cancer) and to test and validate new therapeutic approaches.

## Data Availability

The original contributions presented in the study are included in the article/Supplementary Material, further inquiries can be directed to the corresponding author.
