# Supplementary material for: A miniaturized multicellular platform to mimic the 3D structure of the alveolar-capillary barrier
Source: Front Bioeng Biotechnol. 2024 Apr 5;12:1346660. doi: 10.3389/fbioe.2024.1346660 (PMC11026571; doi:10.3389/fbioe.2024.1346660)
Supplement: Supplementary file 3 [file DataSheet1.docx]

Supplementary Material

**A miniaturized multicellular platform to mimic the 3D structure of the alveolar-capillary barrier**

Michela Licciardello^1,2,3^, Cecilia Traldi^1,2,3^, Martina Cicolini^2,4^, Valentina Bertana^4^, Simone Luigi Marasso^4,5^, Matteo Cocuzza^4^, Chiara Tonda-Turo^1,2,3*†^, Gianluca Ciardelli^1,2,3,6†^

^1^ La.Di.Spe Bioengineerig, Politecnico di Torino, Department of Mechanical and Aerospace Engineering, Turin, Italy

^2^ POLITO BIOMedLAB, Politecnico di Torino, Turin, Italy

^3^ Interuniversity Center for the promotion of the 3Rs principles in teaching and research, Italy

^4^ ChiLab- Materials and Microsystems Laboratory, Politecnico di Torino, Department of Applied Science and Technology (DISAT), Chivasso (TO), Italy

^5^ CNR-IMEM, National Research Council- Institute of Materials for Electronics and Magnetism, Parma, Italy

^6^ CNR-IPCF, National Research Council-Institute for Chemical and Physical Processes, Pisa, Italy

*** Correspondence:**Corresponding Author
[chiara.tondaturo@polito.it](mailto:chiara.tondaturo@polito.it)

^†^ *These authors supervised and contributed equally to this work*

**Supplementary Figures**


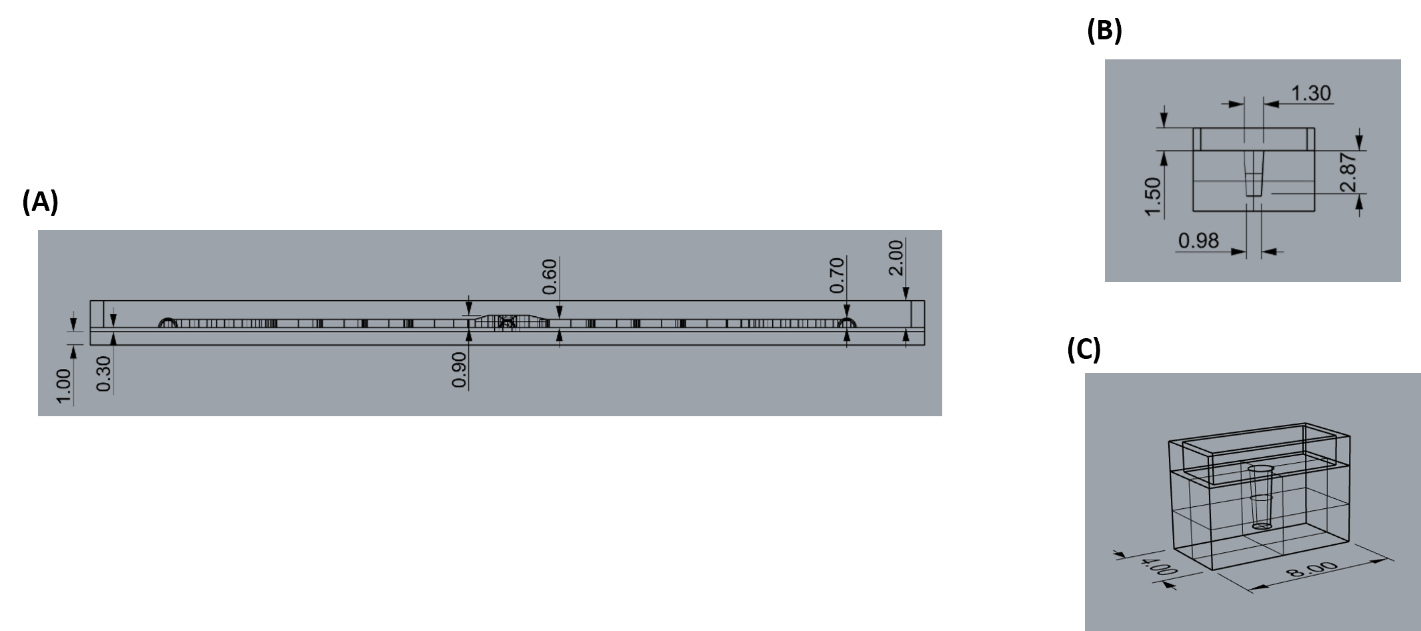


**Supplementary Figure 1.** **CAD drawing of bottom layer** **and lateral plug. (A)** Front view of the CAD model of the bottom layer master. Front view **(B)** and perspective view **(C)** of the CAD model of the lateral plug master. Dimensions are expressed in mm.


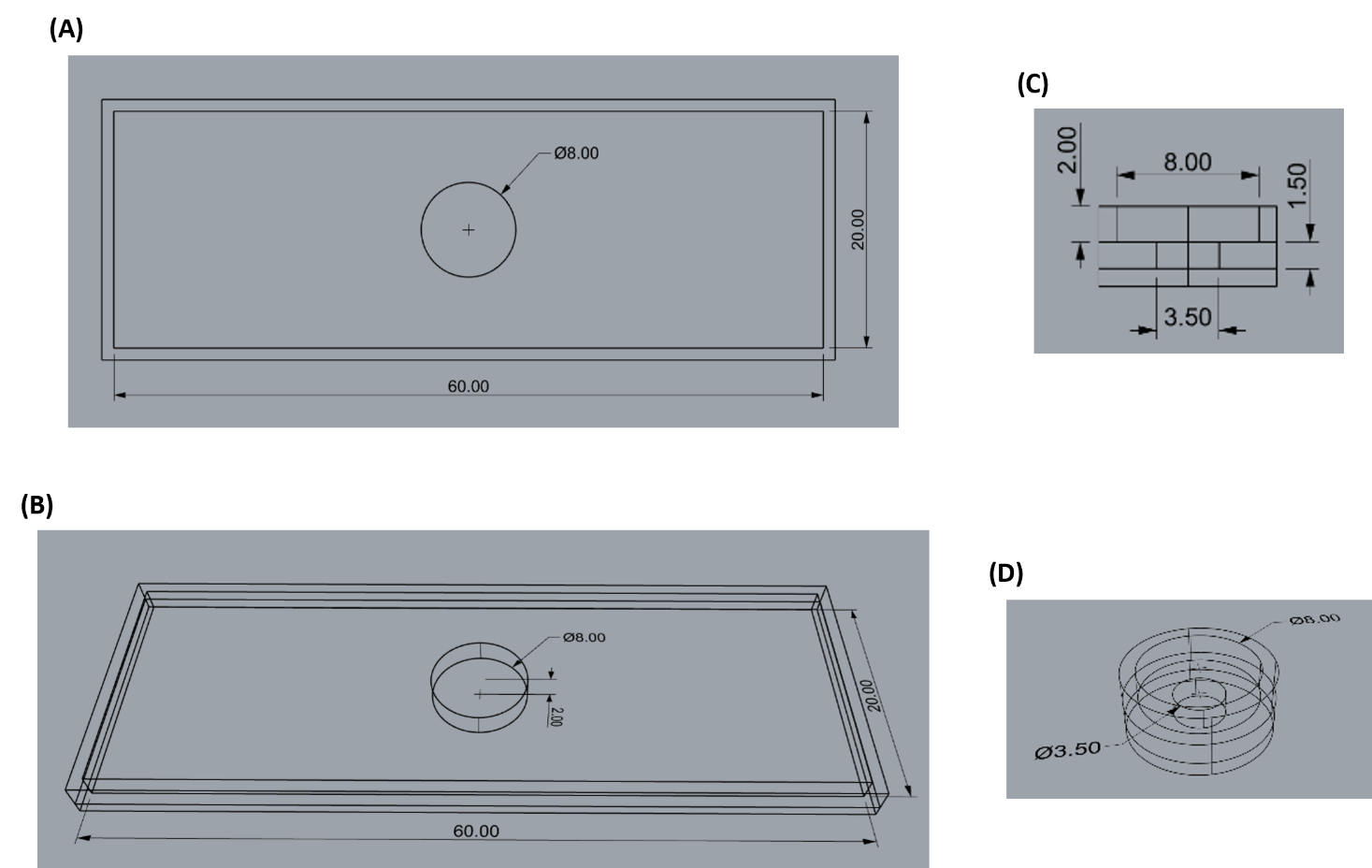


**Supplementary Figure 2. CAD drawing of reservoir layer** **and central plug.** Top view **(A)** and perspective view **(B)** of the CAD model of the reservoir master. Front view **(C)** and perspective view **(D)** of the CAD model of the master of the central plug. Dimensions are expressed in mm.


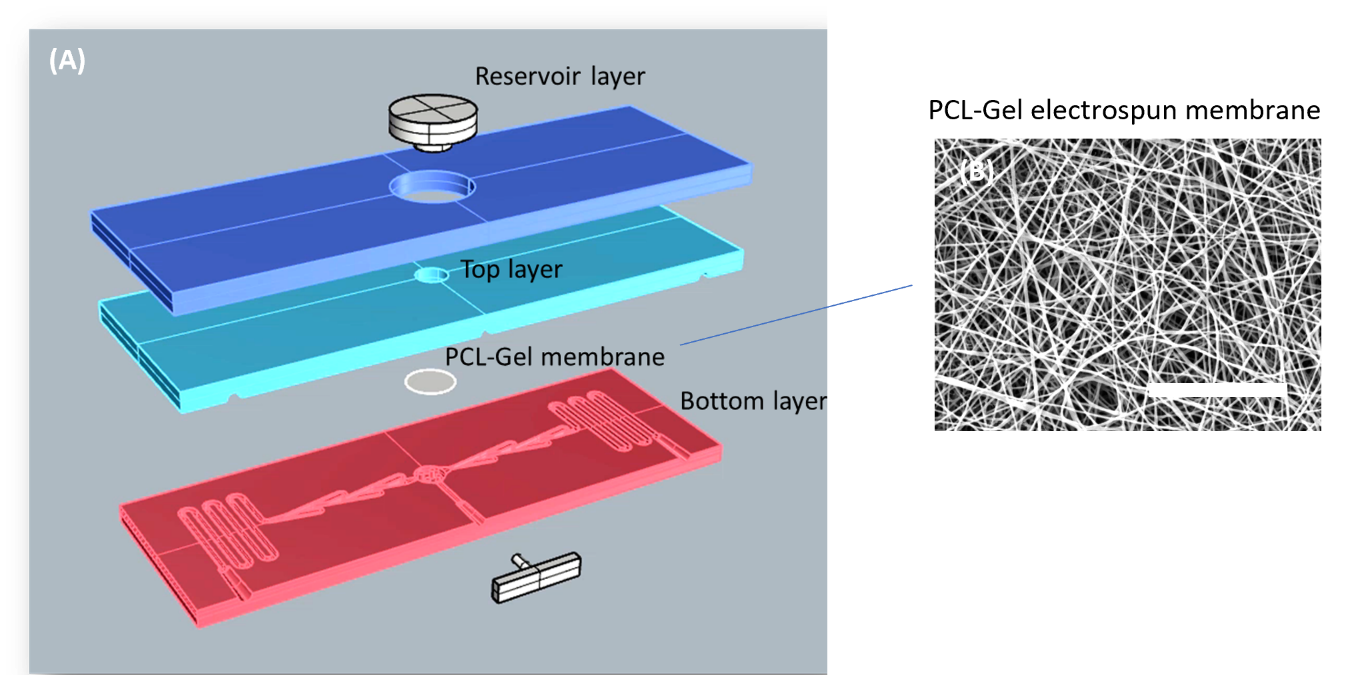


**Supplementary Figure 3. Alveolus-on-a-chip configuration. (A)** Schematic representation of alveolus-on-a-chip configuration. **(B)** Scanning electron microscopy (SEM) image of PCL-Gel electrospun membrane (scale bar = 10 µm).


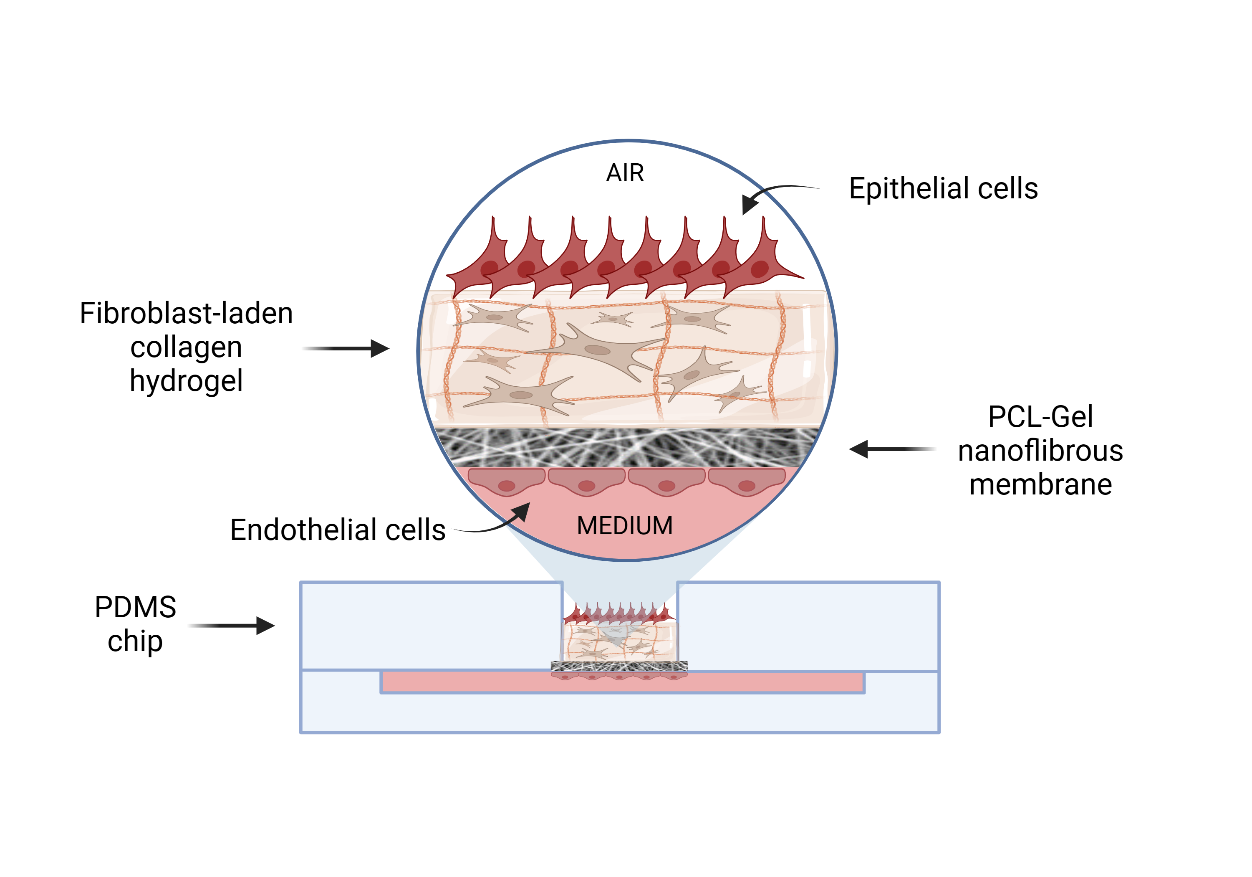


**Supplementary Figure 4. Schematic illustration of the arrangement of the different cell types within the alveolus-on-a-chip.** Endothelial cells (HVEC) are seeded on the bottom side of the PCL-Gel membrane and are located in the basolateral chamber of the device. Fibroblasts (MRC5) embedded in a collagen matrix are seeded in the apical chamber of the chip, on the top side of the PCL-Gel membrane. Epithelial cells (A549) are located on the surface of the MRC5-laden collagen matrix to form a cell monolayer. Created with BioRender.com.


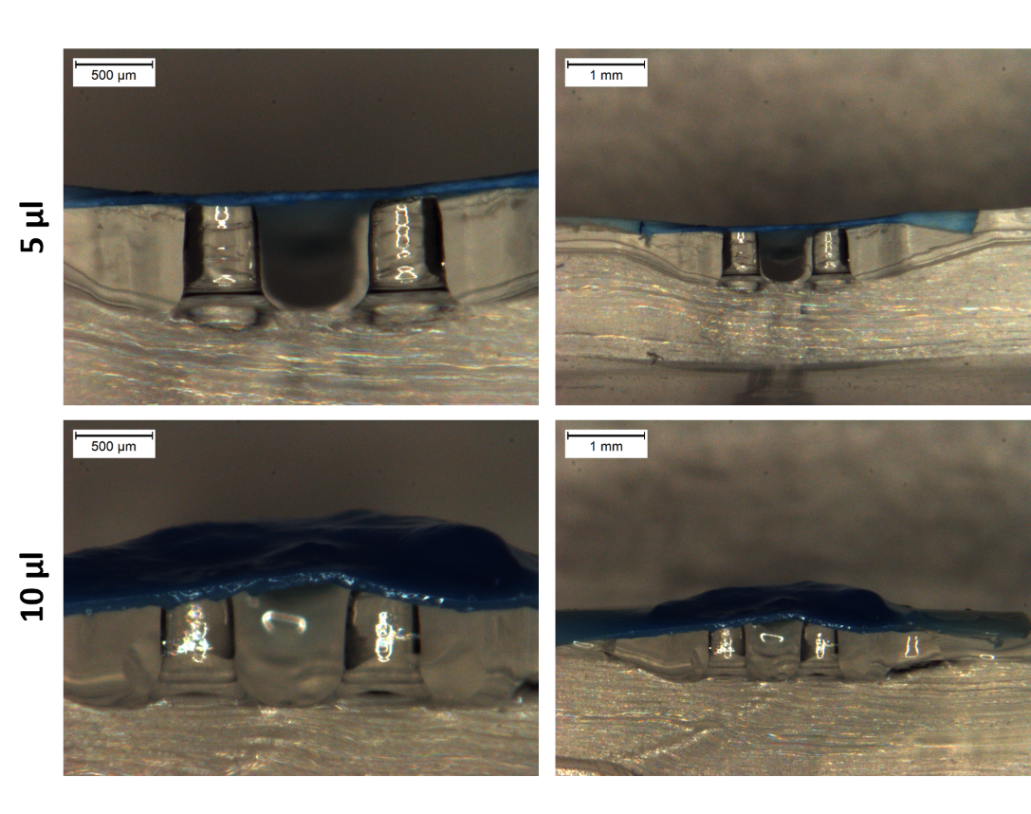


**Supplementary Figure 5. Optimization of collagen hydrogel volume.** Optical images of section of microfluidic device with different volumes (5 µl and 10 µl) of collagen hydrogel. The volume of 5 µl is too low to create a stable hydrogel while with 10 µl a stable depot can be observed atop the PCL/Gel membrane.


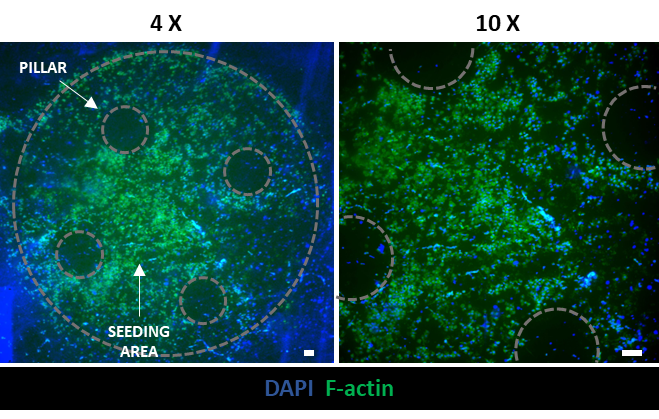


**Supplementary Figure 6. Evaluation of HVEC behavior in the alveolus-on-a-chip.** Representative fluorescence images (n=3) of cytoskeleton staining in HVEC in the basolateral chamber of the chip at 1 day after seeding. (scale bar = 100 μm).


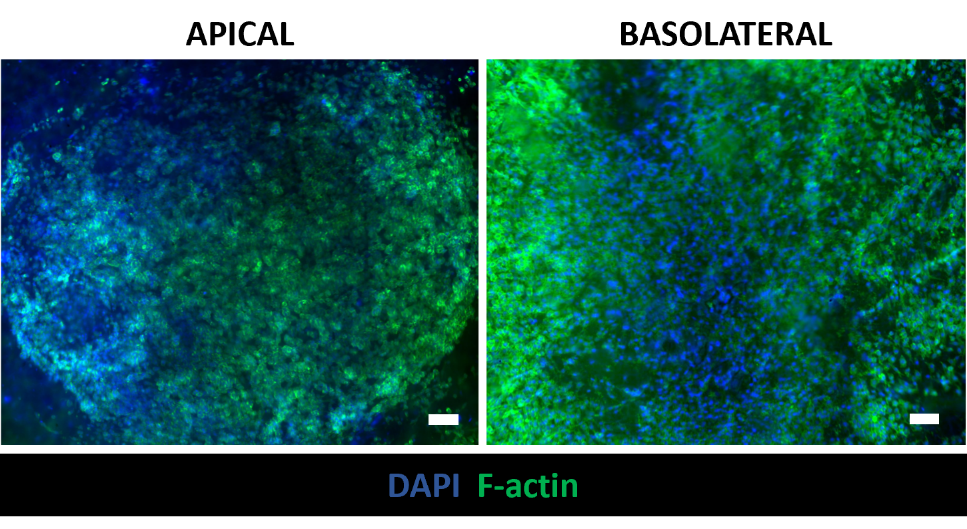


**Supplementary Figure 7. Evaluation of cell behavior in the alveolus-on-a-chip.** Representative fluorescence images (n=3) of cytoskeleton staining in HVEC (in the basolateral chamber), A549 and MRC5 (in the apical chamber), tri-culture after 7 days at ALI. 10 x magnification images (scale bar = 100 μm).


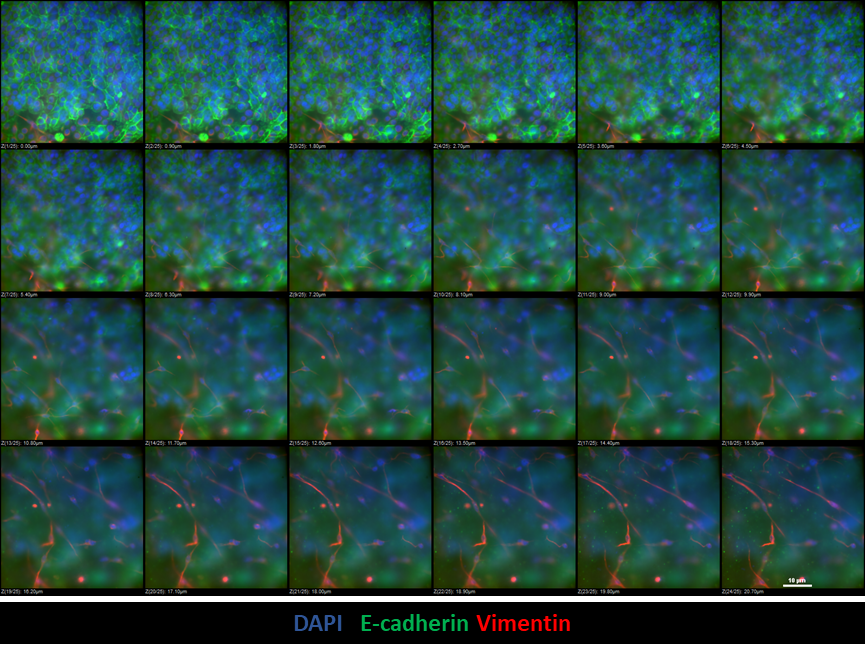


**Supplementary Figure 8. Immunofluorescence staining for E-cadherin and Vimentin in the apical chamber of the alveolus-on-a-chip.** Z-stack image sequence acquired for the apical compartment of the chip. Expression of E-cadherin (green) in A549 cells and Vimentin (red) in MRC-5 fibroblasts after 10 days (7 days at ALI). The images were captured every 0.90 μm for a thickness of around 20 μm. Scale bar = 10 μm.


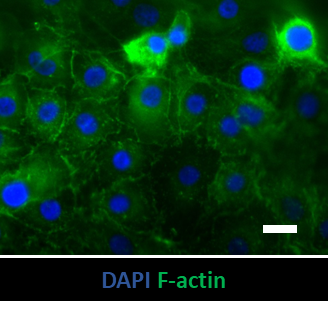


**Supplementary Figure 9. Fluorescence imaging of HVEC cells cultured in the basolateral chamber of the alveolus-on-a-chip.** Representative fluorescence image (n=3) of cytoskeleton staining in HVEC after 10 days (7 days at ALI). Scale bar = 20 μm.


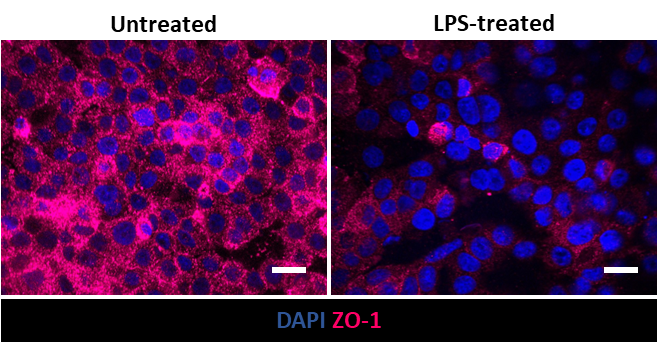


**Supplementary Figure 10.** **Evaluation of cell response to inflammatory insult induced by LPS.** Representative immunofluorescence images (n=3) for ZO-1 in A549 cells cultured in the alveolus-on-a-chip after 2 days of LPS treatment (scale bar = 20 μm).


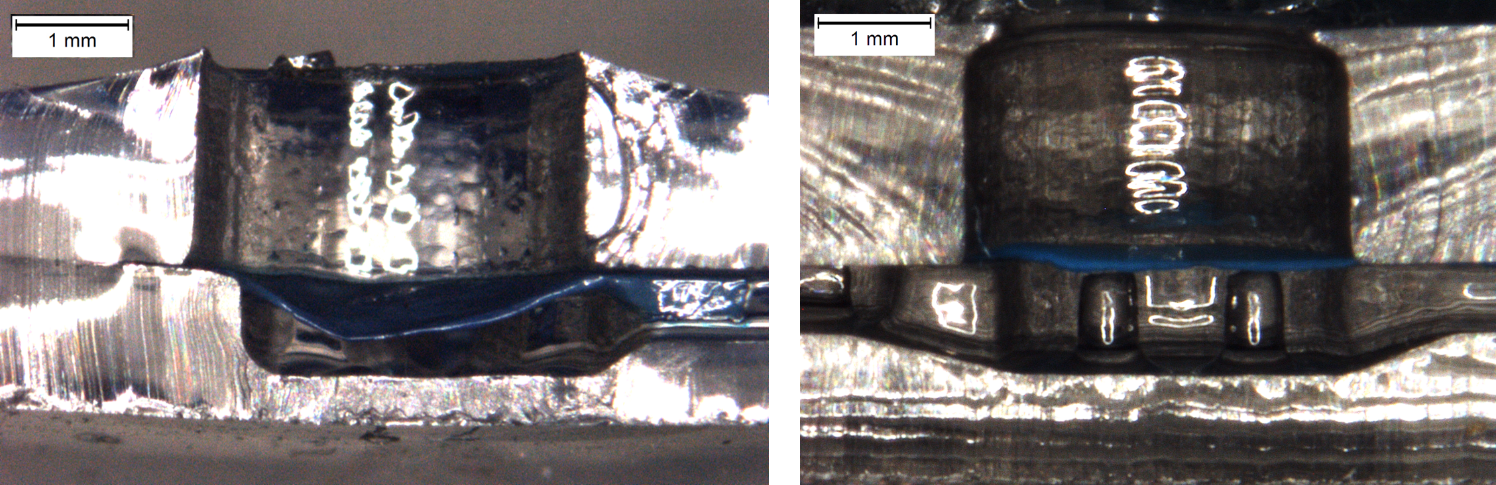


**Supplementary Figure 11. Characterization of the assembled alveolus-on-a-chip.** Optical images of section of microfluidic device without (left) and with (right) pillars.
